# Correlation Between Ankle Brachial Index and Lower Limbs Digital Pulse Oximetry: A Referral Center Experience, Prevalence Study

**DOI:** 10.7759/cureus.6762

**Published:** 2020-01-24

**Authors:** Juan Sanjuan, Edwin Romero, Rolando Medina, Wilmer Botache, Gabriela Ruiz, Andres Ramirez, Estefania Barbosa, Maria Andrade, Roberto Diaz, Francisco J Montoya

**Affiliations:** 1 Clinical Research, Utopiapp SAS, Cali, COL; 2 Surgery, Universidad Surcolombiana, Neiva, COL; 3 General Surgery, Universidad Surcolombiana, Neiva, COL

**Keywords:** lower limbs, atherosclerosis, ankle-brachial index, peripheral vascular disease

## Abstract

Objective

Ankle-brachial index (ABI) is widely recommended and used to evaluate peripheral arterial disease. The oxygen saturation (SpO_2_) has been associated with ABI, showing a promising clinical practice utility; however, little literature regarding this matter has been reported. This study aims to assess the correlation between pulse oximetry and ABI.

Methods

A cross-sectional study was conducted using ABI measuring devices, such as the MESI® Ankle Brachial Pressure Index (ABPI) (MESI, Ltd., Slovenia, EU) and pulse oximetry. We compared the SpO_2_ distribution by using the Wilcoxon test and evaluated its correlation by using logistic regression.

Results

From a total of 86 patients, 54 were males (62.8%) and the median age was 54 years old (interquartile range (IQR) = 37 - 65 yrs.). Regarding ABI measurements of the right lower limb (RLL), a total of 20 patients (22.3%) had an abnormal classification. On the other hand, a total of 21 patients (22.1%) had an abnormal classification of the left lower limb (LLL) ABI measurements. The distribution of SpO_2_ in relation to ABI categories was not statistically different (RLL p = 0.2433; LLL p = 0.1242). The SpO_2_ classification of ABI and abnormal pulse oximetry for the RLL was at 76.7% and at 77.9% in the LLL (Pearson’s goodness-of-fit test: RLL = p < 0.001 and LLL = p < 0.001).

Conclusion

Although we didn’t observe any statistical differences in the SpO_2_ distribution regarding ABI measurements, in their correlation, there seems to be a different tendency. The SpO_2_ might be a useful non-invasive tool to assess asymptomatic patients with risk factors for peripheral arterial disease (PAD).

## Introduction

Ankle-brachial index (ABI) is the ratio of the systolic blood pressures of the ankles and arms. It is one of the most useful tests for identifying peripheral arterial disease (PAD) because it can be easily applied, is simple, cheap, and non-invasive. In regard to PAD, the test accuracy is 86% - 99% and its sensibility is 75% - 95%. PAD is often described as a decrease of blood supply to the lower limbs because of an obstruction narrowing the vessel walls, thus reducing blood flow, generally due to atherosclerosis [1-2]. This disease affects over 202 million people worldwide, and it seems to be an independent indicator of cardiovascular morbidity and mortality. Furthermore, it is important for coronary history since patients with PAD showed more risk for both short and long-term coronary artery disease, stroke, myocardial infarction, limb ischemia, and death [3-4]. Although ABI has great prognostic and diagnostic value, it also has several limitations, such as gender, age, calf size, daily stress, sphygmomanometer size, its position, etc. However, it remains an important tool in practice [5-7].

The use of oxygen saturation (SpO_2_) is a common tool used during physical examinations as this non-invasive technique is easy and inexpensive to perform [8]. Some experiences describe a certain relationship between pulse oximetry and ABI in the diagnosis of lower extremity arterial disease [9]. The measurements represent blood flow and SpO_2_ and are also used as an evaluation after reperfusion injury [9]. In the literature, the ABI relationship and a SpO_2_ variation of 2% - 5% have been suggested as an effective additional method for screening patients with PAD [9-11]. This relationship remains unclear considering the interacting factors [12-13]. Our aim was to assess the correlation between pulse oximetry and ABI in an academic referral hospital setting.

## Materials and methods

We conducted a cross-sectional study between the months of April and June of 2017 in non-critical care units of an academic referral hospital in South Colombia. A non-probabilistic convenience sample size was considered. Additionally, patients with a blood pressure measurement contraindications were excluded. Throughout this study, we used non-invasive ABI measuring devices, such as the MESI® Ankle Brachial Pressure Index (ABPI) (MESI, Ltd., Slovenia, EU) and pulse oximetry, such as Vismo® (Vismo, York, UK). First, we collected the demographics and comorbidity information of the patients. Afterward, we explained the informed consent act and obtained patient consent. Later on, we carried out a general clinical evaluation and performed the ABI and SpO_2_ measurements. This study was approved by the Institutional Review Board of the Hospital Universitario Hernando Moncaleano Perdomo de Neiva.

Definitions

Abnormal pulse oximetry was defined as > 2% = SpO_2_’s upper limb and SpO_2_’s ipsilateral lower limb [13]. ABI categories were defined according to a measuring index. The reference measuring index is between ≥ 0.91 and < 1.3, an abnormal one is between ≥ 0.41 and < 0.91, a critical one is between ≥ 0 and < 0.41, and a subnormal one is ≥ 1.30 [14]. 

Statistical analysis

Statistical analyses were performed using Stata®, version 15 (StataCorp LLC, College Station, Texas, USA). A descriptive statistic was performed which made use of absolute (N) and relative (%) frequencies for categorical variables. For continuous variables, median (Med) and interquartile range (IQR) were employed. In order to compare SpO_2_'s distribution, the Wilcoxon test was applied which showed a significant correlation of p-value < 0.05 between abnormal pulse oximetry and ABI. This correlation was assessed by using logistic regression with a 95% confidence interval, a significant p-value < 0.05, and the Pearson goodness-of-fit test post-estimation.

## Results

From a total of 86 patients that were included in the study, 54 were male (62.8%), and the median age was 54 years old (IQR: 37 - 65 yrs). The most frequent insurance type among patients was subsidized affiliation which covered 46 patients (54.5%). The second most frequent insurance type was a contributive affiliation which covered 24 patients (27.9%). The most frequent comorbidities were hypertension and diabetes (Table 1). Likewise, 23 patients (26.7%) declared having a smoking habit in their case histories. Previous cases of outpatient medication involving beta-blockers, statins, and antiplatelet drugs showed low frequency (Table 1).

**Table 1:** Sociodemographic Characteristics of Patients Evaluated with ABI and SpO2 ABI: ankle-brachial index; SpO_2_: peripheral capillary oxygen saturation * Median (interquartile range)

	Total n (%)	
Age (years)*	54	(37 - 65)
Male	54	(62.8)
Insurance		
Subsidized	46	(53.5)
Contributive	24	(27.9)
Special	11	(12.8)
No coverage	4	(4.7)
Comorbidities		
Diabetes mellitus	18	(20.9)
Smoking	23	(26.7)
High blood pressure	26	(30.2)
Stroke	5	(5.8)
Premedication		
Acetylsalicylic acid	9	(10.5)
Beta-blockers	4	(4.7)
Statins	6	(7.0)

**Figure 1:**
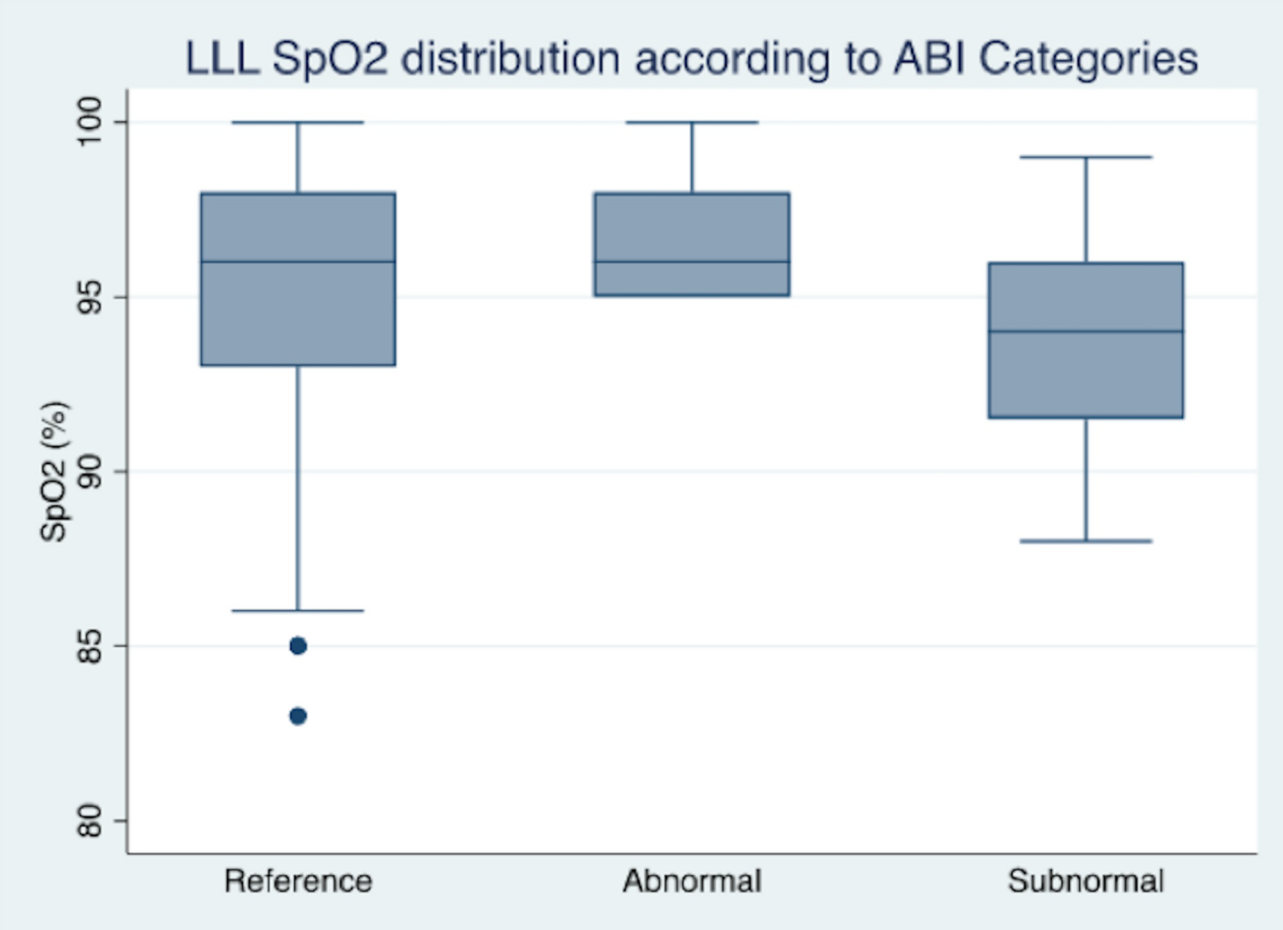
Graph box of SpO2 distribution according to LLL ABI reference of patients evaluated with ABI and SpO2 ABI: ankle-brachial index; LLL: left lower limb; SpO_2_: peripheral capillary oxygen saturation

**Figure 2:**
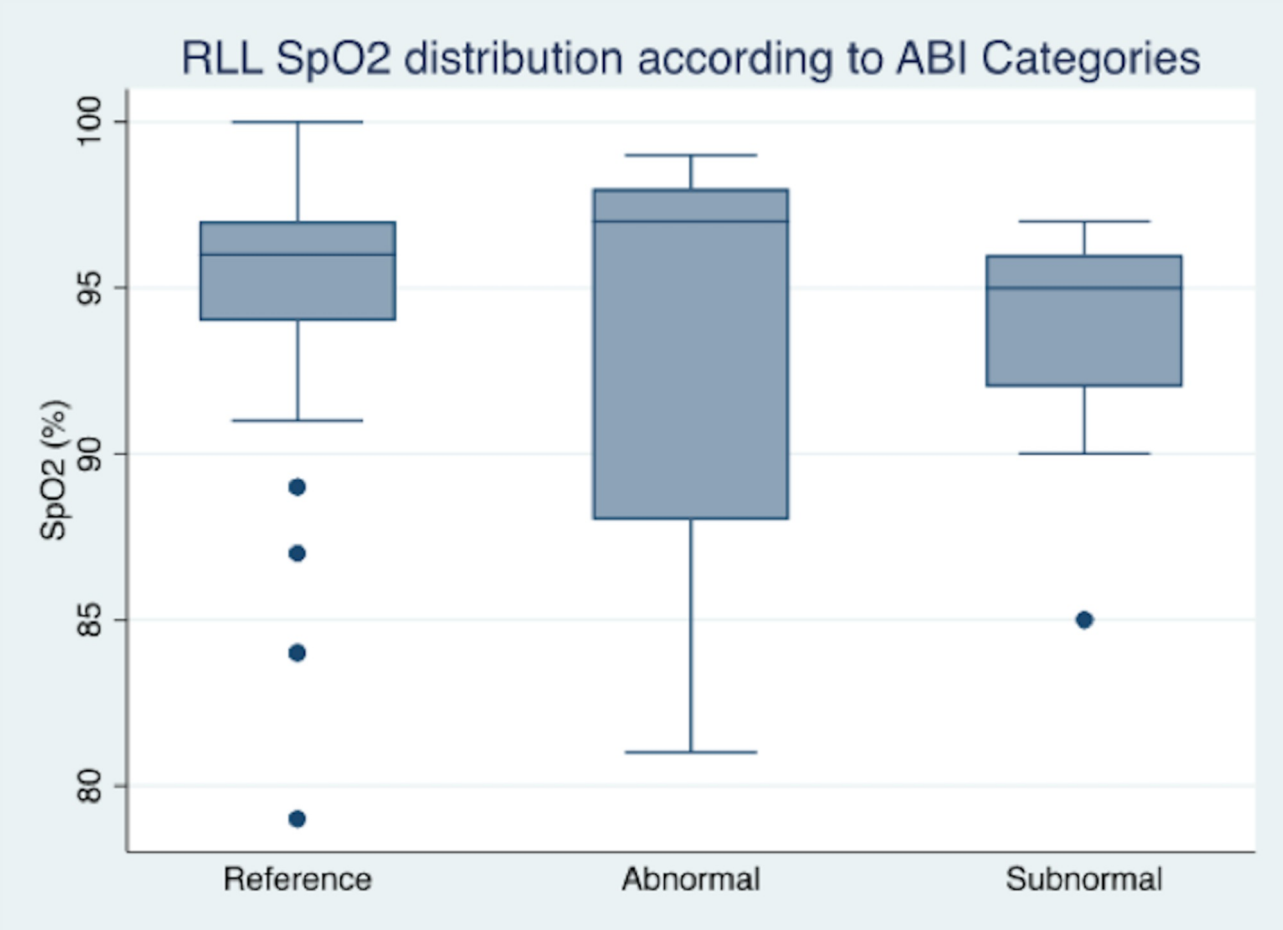
Graph box of SpO2 distribution according to the RLL ABI reference of patients evaluated with ABI and SpO2 ABI: ankle-brachial index; RLL: right lower limb; SpO_2_: peripheral capillary oxygen saturation

In regard to the right lower limb (RLL) ABI measurements, a total of 66 patients (77.7%) were classified in the reference category, while 20 patients (22.3%) were classified as abnormal. In regard to the ABI measurements of the left lower limb (LLL), a total of 67 patients (77.9%) were classified in the reference category, whereas 21 patients (22.1%) had an abnormal classification. The median SpO_2_ distribution of the RLL reference category was 96% (IQR: 94% - 97%) and was 96% in the LLL reference category (IQR: 94% - 97%) (Figures 1-2). No statistical difference was found within the ABI categories (RLL: p-value = 0.2433; LLL: p-value = 0.1242); this statistical comparison was not as significant as expected, despite comparing the reference group to non-reference measurements (RLL: p-value = 0.2126; LLL: p-value = 0.4293). The SpO_2_ versus ABI and abnormal pulse oximetry in RLL were correctly classified in 76.7% and 77.9% in the LLL (Pearson’s goodness-of-fit test RLL: p-value < 0.001 and LLL: p-value < 0.001) (Table 2).

**Table 2:** SpO2 Distribution According to ABI Reference of Patients Evaluated with ABI and SpO2 ABI: ankle-brachial index; IQR: interquartile range; Med: median; RLL: right lower limb; LLL: left lower limb; SpO_2_: peripheral capillary oxygen saturation

			n		(%)	Med (IQR)			p-value		n	(%)			Med (IQR)		p-value	
							RLL							LLL				
Reference		66		(77.7)			96 (94-97)	0.2433		67			(77.9)	96 (94-97)		0.1242		
Abnormal		6		(7.0)			97 (88-98)			7			(8.1)	97 (88-98)				
Critical		.		.			.			.			.	.				
Subnormal		14		(16.3)			95 (92-96)			12			(14.0)	95 (92-96)				

## Discussion

Patients with PAD have a higher functional impairment which may lead to amputations. Losing such clinical and functional life quality could turn out as an economic burden; in fact, it is often an under-recognized or underestimated entity during clinical practice [15-17]. In the literature, the most recommended non-invasive measure is ABI, which can be performed through Doppler and blood pressure readings [1-2]. In this standard practice, the patient is required to lay down in the supine position with his/her legs and heart horizontally (at the same height) so that the pulse rate of either the dorsalis pedis or tibial posterior artery can be checked. The higher systolic blood pressure (SBP) from the ankles is divided between the major SBP of the arm [18]. According to some studies, the ABI measurement has been recommended to be performed in asymptomatic patients between the ages of 50 to 65 years with PAD risk factors or clinical suspicion of it [7, 19]. In regards to our experience, most of the patients were younger than 65 years old and had a low atherosclerosis probability. This could also explain why there were no critical observations in ABI classifications [20-22]. Our study population consisted of patients mostly under the age of 65 years and patients who had subsidized insurance (this refers to people with unstable or no economic income). 

Although we did not observe any statistical differences in SpO_2_ distribution measurements among ABI categories, the box plots showed a tendency towards a lower distribution within the non-reference classification. This result is more related to the specificity of ABI and SpO_2_ than it is to the sensitivity classification. According to the purpose of this study, claudication symptoms were not taken into account as they show asymptomatic patients who never had a PAD history. The observed SpO_2_ distribution behavior was consistent with the literature, which simultaneously added more information to this subject. The SpO_2_ measurement value was assessed with a 2% - 5% difference; throughout our analysis, no statistical differences were found regarding finger-toe subtraction or the area under a curve near 60% with a non-significant goodness-of-fit test. This could elaborate more on the correlation between these findings; however, sample size hampers the analysis as described in other experiences [10-11, 13]. These results from the clinical setting may suggest that SpO_2 _can be a useful, non-invasive tool for assessing asymptomatic patients who are prone to PAD risk and have any blood pressure measuring contraindications. Our experience relays more data to this research line, and these findings are consistent with other experiences and relate to other invasive or treatment procedures [10-11, 13]. We also like to mention that additional to SpO_2_, the transcutaneous partial pressure of oxygen (TcPO_2_) is another vascular tool commonly used in vascular medicine to reflect local arterial blood flow and skin oxygenation. This tool has been described as a potential predictor of cardiovascular events and blood flow before and after revascularization in patients with peripheral artery disease, including in patients with conditions such as end-stage renal disease and diabetes [23-25].

As was previously mentioned, this study is limited by sample size, which might affect the significance and performance of the statistical tests through factors, such as lacking critical patients classified according to ABI. Furthermore, when interpreting these results, an explanation for the SpO_2_ correlation was uncovered. Another consideration for the interpretation of our results related to the performance of this correlation when classified as normal. This consideration is based on the heterogeneity recognition of categories that suggest the involvement of peripheral arterial disease and/or subnormal category when the ABI is > 1.3. We believe that the correlation may vary under the conditions of low hemoglobin levels or other systemic perfusion alterations that might be heterogeneous in SpO_2_ distribution. The correlation assessment of both variables was evaluated with logistic regression as far as was feasible. We feel this assessment might contribute to the estimations to come regarding this issue. However, it should be noted that this can also act as a limitation to residual treatment due to the limited sample size. Similarly, as described in other experiences, we identified the need for doing further research regarding the relationship between SpO_2_ and ABI within the PAD context.

## Conclusions

Although we didn’t observe any statistical differences in SpO_2_ distribution regarding ABI measurements, SpO_2_ can be a useful non-invasive tool to assess asymptomatic patients with risk factors for peripheral arterial disease (PAD).
